# Rapid Detection of *Brettanomyces bruxellensis* in Wine by Polychromatic Flow Cytometry

**DOI:** 10.3390/ijms232315091

**Published:** 2022-12-01

**Authors:** Domenico De Bellis, Alessio Di Stefano, Pasquale Simeone, Giulia Catitti, Simone Vespa, Antonia Patruno, Marco Marchisio, Eleonora Mari, Lisa Granchi, Carlo Viti, Piero Chiacchiaretta, Angelo Cichelli, Rosanna Tofalo, Paola Lanuti

**Affiliations:** 1 Department of Medicine and Aging Sciences, University “G. d’Annunzio” of Chieti-Pescara, 66100 Chieti, Italy; 2 Center for Advanced Studies and Technology (CAST), University “G. d’Annunzio” of Chieti-Pescara, 66100 Chieti, Italy; 3 FlowForLife Lab, Spin-Off, Center for Advanced Studies and Technology (CAST), University “G. d’Annunzio” of Chieti-Pescara, 66100 Chieti, Italy; 4 Department of Agronomy, Food, Environmental and Forestry, University of Florence, Piazzale delle Cascine 18, 50144 Firenze, Italy; 5 Department of Innovative Technologies in Medicine and Dentistry, University “G. d’Annunzio” of Chieti-Pescara, 66100 Chieti, Italy; 6 Advanced Computing Core, Center for Advanced Studies and Technology (CAST), University “G. d’Annunzio” of Chieti–Pescara, Via Luigi Polacchi 11, 66100 Chieti, Italy; 7 Department of Bioscience and Technology for Food, Agriculture and Environment, University of Teramo, Via R. Balzarini 1, 64100 Teramo, Italy

**Keywords:** *Brettanomyces bruxellensis*, flow cytometry, wine contamination

## Abstract

*Brettanomyces bruxellensis* is found in several fermented matrices and produces relevant alterations to the wine quality. The methods usually used to identify *B. bruxellensis* contamination are based on conventional microbiological techniques that require long procedures (15 days), causing the yeast to spread in the meantime. Recently, a flow cytometry kit for the rapid detection (1–2 h) of *B. bruxellensis* in wine has been developed. The feasibility of the method was assessed in a synthetic medium as well as in wine samples by detecting *B. bruxellensis* in the presence of other yeast species (*Saccharomyces cerevisiae* and *Pichia* spp.) and at the concentrations that produce natural contaminations (up to 10^5^ cells/mL), as well as at lower concentrations (10^3^–10^2^ cells/mL). Wine samples naturally contaminated by *B. bruxellensis* or inoculated with four different strains of *B. bruxellensis* species together with *Saccharomyces cerevisiae* and *Pichia* spp., were analyzed by flow cytometry. Plate counts were carried out in parallel to flow cytometry. We provide evidence that flow cytometry allows the rapid detection of *B. bruxellensis* in simple and complex mixtures. Therefore, this technique has great potential for the detection of *B. bruxellensis* and could allow preventive actions to reduce wine spoilage.

## 1. Introduction

*Brettanomyces bruxellensis* is an oval/ellipsoidal yeast 2–7 µm in diameter [1], growing in fermented beverages, such as wine, beer, and cider [1,2]. *Dekkera*/*Brettanomyces* genus, belonging to the Pichiaceae family, comprises five anamorphic species (*B. custersianus*, *B. naardenesis*, *B. nanus*, *B. anomalous*, and *B. bruxellensis*) and two teleomorphic forms (*D. anomala* and D. *bruxellensis*) [3]. Its genus was recognized in the 1920s, when it was isolated from a pool of yeasts obtained from the Belgian “Lambic ale” beer [4]. In the 1950s and 1960s, the yeast was identified in wine, for the first time in France, Italy, and South Africa, and further in other countries. However, studies on *B. bruxellensis* increased later, over the 1980s and 1990s [4,5]. Only *B. bruxellensis* strains are able to release detrimental compounds, e.g., volatile phenols (e.g., 4-ethyl phenol and 4-ethyl guaiacol) derived from the sequential conversion of hydroxycinnamic acids and *p*-coumarate. These molecules confer to wines the so-called “Brett-character”, linked to a bad smell aroma, which leads to consequent qualitative and economic problems [6,7,8]. Frequently, *B. bruxellensis* is also found in cellar equipment, often when the cleaning processes are not effective [6]. *B. bruxellensis* survives even for a long time, especially in wooden barrels [9]. In these cases, *B. bruxellensis* penetrates into the barrique staves (up to 8 mm of depth) [10], making the sanitization procedures useless. Of note, *B. bruxellensis* tolerates ethanol, as well as low pH levels and its growth is stimulated by oxygen [11,12]. Therefore, the oxygen penetration through the wood and the practice of micro-oxygenation, which allows for ethanol oxidation and the formation of a more stable bond between tannins and anthocyanins, crucial for the stabilization of the red wine color [13], are conditions that promote *B. bruxellensis* development. It has been demonstrated that *B. bruxellensis* also affects the wine color [14], hydrolyzing the anthocyanins and producing a color loss [6,9]. Overall, the presence of *B. bruxellensis* during the fermentation processes produces relevant alterations to the wine sensory profile. *B. bruxellensis* is able to produce 4-vinyl phenol and 4-ethyl phenol, which can contribute to wine bouquet complexity when present in a low concentration. However, above the sensory threshold they confer off-flavors described as “horse sweat”, “medicinal”, “smoke”, “phenolic”, “barnyard”, “rancid”, and “sweaty”. Moreover, these yeasts can have other detrimental effects, such as the production of biogenic amines, tetrahydropyridines from lysine (responsible for a mousy “off-flavor”), acetic acid, nonenal, guaiacol and several ethyl-esters from short-chain fatty acids [15]. For these reasons, the possibility to identify *B. bruxellensis* contaminations within the barrels or during the first fermentation phases may allow for the adoption of appropriate strategies to avoid the production of altered wines. The methods traditionally used to detect such yeast during wine production are mainly based on the application of conventional microbiological techniques and need up to 15 days [1,16]. In addition, before identifying the contamination, the yeast can grow and spread, causing significant spoilage in wines. Moreover, PCR-based methods (e.g., Real-Time PCR protocols) have been developed [17]. The reverse transcriptase PCR (RT-PCR) allows for discriminating between viable and non-viable cells, while nested-PCR allows for the direct detection of *B. bruxellensis* in wines without strain isolation [9,18,19]. Even if these molecular methods are faster than plate count techniques, they do not allow for yeast count and are particularly expensive. Impedance has also been used for the detection of *B. bruxellensis* [20]. Besides the rapidity of the execution of this method, it is not specific. Recently, a flow cytometry kit for the identification and quantification of viable Brettanomyces yeasts in wines (Kit Bretta Test 80 tests, B80, Amarok Biotechnologies, Saint-Malo, France) in a short time (1–2 h) was developed. There are no reports in the literature evidencing the suitability and effectiveness of this method. For this reason, the kit was validated, for the first time using wine samples artificially inoculated or naturally contaminated by *B. bruxellensis*.

## 2. Results

### 2.1. Specificity of the Flow Cytometry Measurements

**Figure 1:**
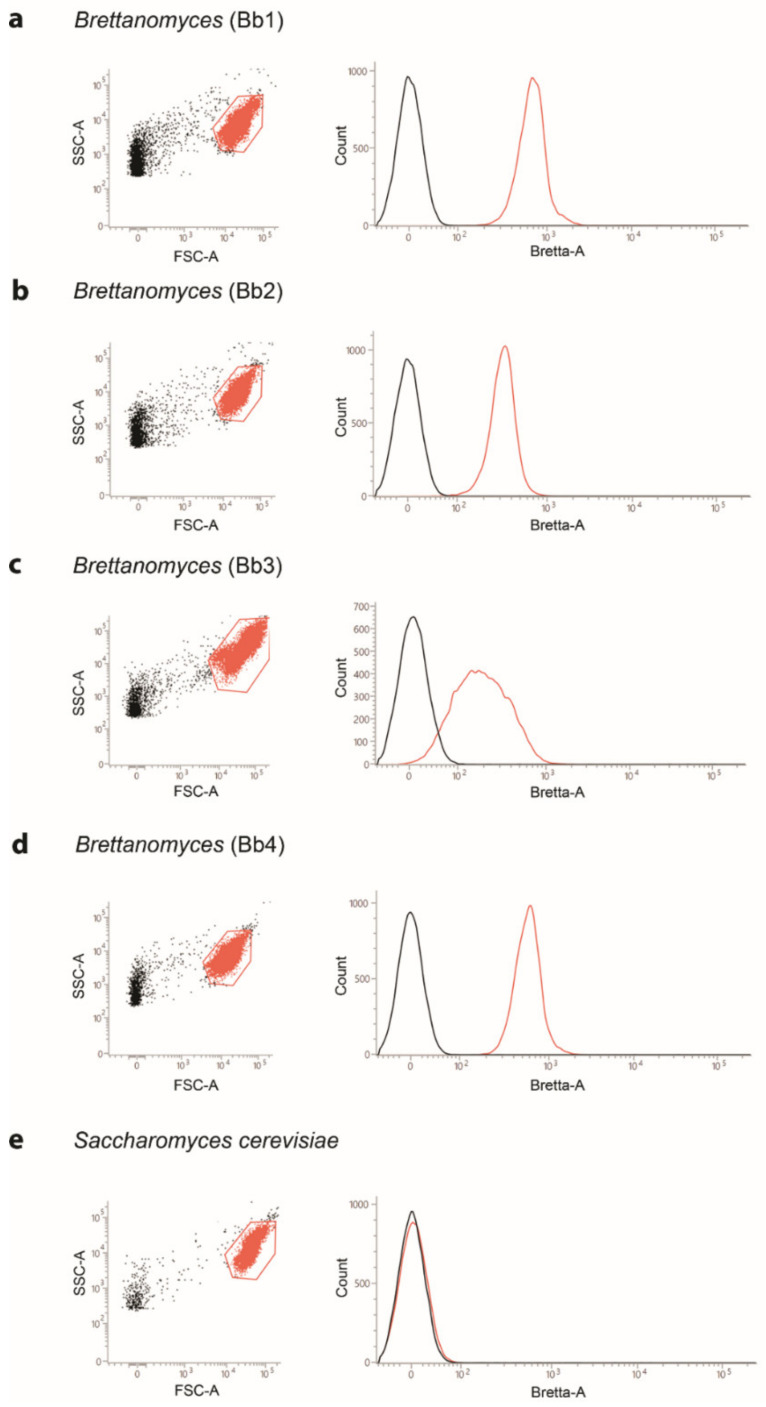
Flow cytometry detection of different *B. bruxellensis* strains. (**a**–**d**) Four different *B. bruxellensis* strains were stained for flow cytometry analyses (Bb1, Bb2, Bb3, Bb4). For all strains, a morphologically homogeneous population (orange dots) of cells was identified on an FSC-A/SSC-A dot-plot (left images). Those cells were analyzed for the expression of *B. bruxellensis* antigens on left histograms (black curves represent the unstained controls, while the related stained samples were overlaid as red curves); (**e**) A sample of *S. cerevisiae* was used as a negative control.

The Bretta Test (Kit Bretta Test 80 tests, B80, Amarok Biotechnologies, Saint-Malo, France) consists of a probe, the fluorescein diacetate (FDA), able to stain viable and metabolically active cells [21,22], as well as a rabbit polyclonal antibody, recognizing Brettanomyces antigens. As a first step, we tested the ability of the antibody to recognize different *B. bruxellensis* strains (Bb1, Bb2, Bb3, Bb4) in pure cultures and at different concentrations. As shown in Figure 1, when cells were analyzed by flow cytometry, a morphologically homogeneous population of yeasts was identified with respect to the scatter parameters (FSC-A/SSC-A, Figure 1a–d, left) for all the tested strains. This population was analyzed for the expression of *B. bruxellensis* antigens, and, as it is shown in the histograms (Figure 1a–d, right), 100% of all *B. bruxellensis* analyzed strains were recognized by the anti-Bretta antibody. Of note, when a sample of *S. cerevisiae* was analyzed, no fluorescence was evidenced in the channel used for the detection of specific *B. bruxellensis* antigens (Figure 1e, right). When higher concentrations of *B. bruxellensis* were analyzed (10^8^, 10^7^ and 10^6^ cells/mL), the staining resulted weaker than with lower concentrations, suggesting that the kit was developed to stain *B. bruxellensis* at concentrations that produce natural contaminations (up to 10^5^ cells/mL). Therefore, when samples containing higher *B. bruxellensis* concentrations are analyzed (10^6^–10^8^ cells/mL), the primary antibody must be titrated under the assay conditions, as recommended [23].

### 2.2. Sensitivity of B. bruxellensis Flow Cytometry Measurements

**Figure 2:**
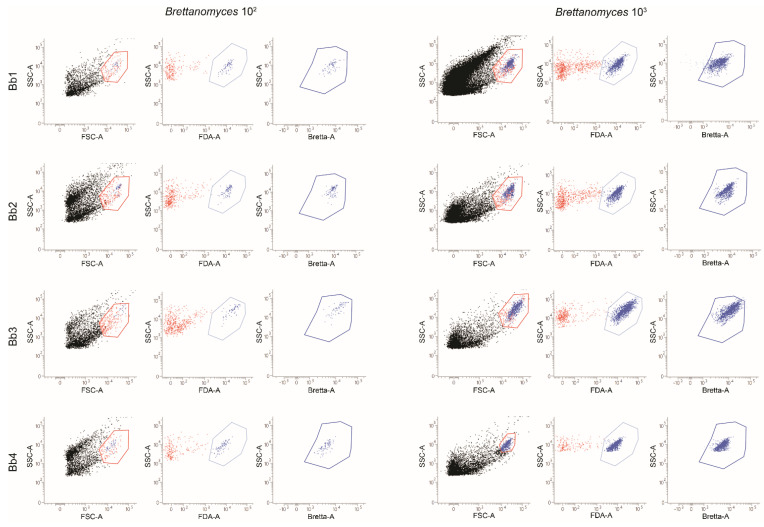
Flow cytometry detection of different *B. bruxellensis* concentrations (10^3^ and 10^2^ cell/mL) from pure cultures. The same *B. bruxellensis* strains reported in Figure 1 were analyzed at lower concentrations (10^3^ and 10^2^ cells/mL). The gating strategy used for the count is shown here: the homogeneous population of yeasts on the FSC-A/SSC-A dot-plot was gated (orange region) and shown on an FDA-A/SSC-A dot-plot. Events positive to the FDA staining (light blue gate) were identified as metabolically active yeasts and finally plotted on a dot-plot showing the staining of the *B. bruxellensis* specific antigens (blue gate). Data are representative of three separate experiments.

To verify the ability of the flow cytometry method to identify *B. bruxellensis* levels compatible with those detectable in cellars, samples of *B. bruxellensis* at lower concentrations (10^3^ and 10^2^ cells/mL) were analyzed (Figure 2). As shown, for all analyzed *B. bruxellensis* strains, a population of yeasts was identified on the dot-plot displaying the morphological parameters (FSC-A/SSC-A dot-plots) that stained positive for the FDA and showed a significant expression of *B. bruxellensis* antigens. As evidenced in Figure 2, both 10^2^ and10^3^ concentrations of all analyzed *B. bruxellensis* strains were detected.

### 2.3. Flow Cytometry Analyses of S. cerevisiae and B. bruxellensis Mixed Cultures

**Figure 3:**
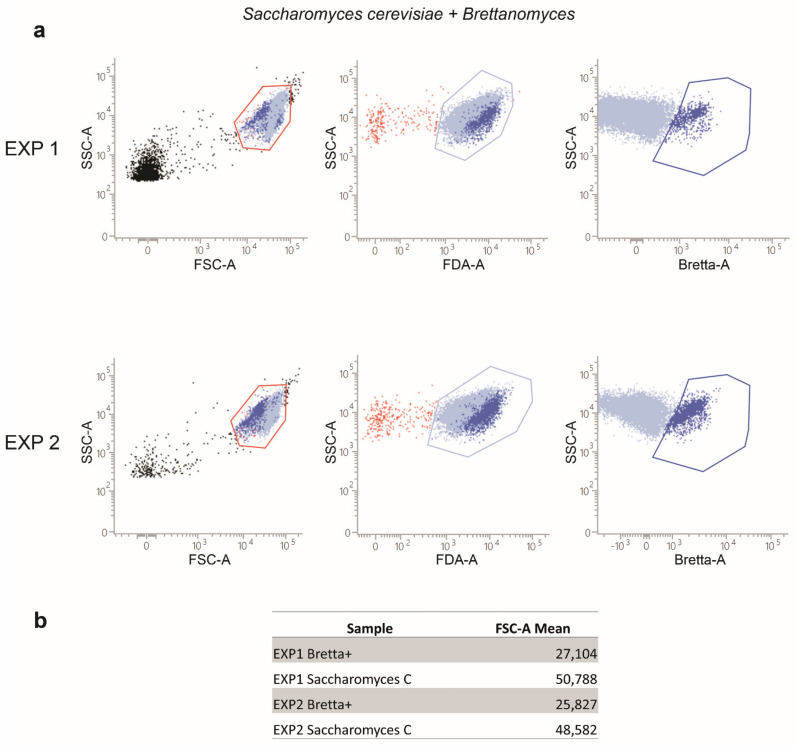
Flow cytometry detection of *B. bruxellensis* in mixed cultures containing *B. bruxellensis* and *S. cerevisiae*. (**a**) Dot-plots, refer to two different separate experiments (EXP1 and EXP2). The gating strategy is shown for both experiments: on a dot-plot FSC-A/SSC-A the yeast populations were morphologically identified, then yeasts positive to the FDA were selected (light blue gates) and metabolically active yeasts were analyzed for the *B. bruxellensis* antigens. (**b**) The table shows the FSC-A Mean Fluorescence Intensity values, both for *B. bruxellensis* (Bretta +) and for *S. cerevisiae*.

Given that wines are complex mixtures containing different types of micro-organisms, we also tested the ability of such a flow cytometry method to identify *B. bruxellensis* (Kit Bretta Test 80 tests, B80, Amarok Biotechnologies, Saint-Malo, France) in samples where different concentrations of *S. cerevisiae* were also inoculated. Figure 3 shows that *B. bruxellensis* was identified by the flow cytometry positivity to the anti-Brettanomyces. antibody provided by the kit. Here, the whole population of yeasts was first identified by their morphological parameters (FSC-A/SSC-A), then FDA+ cells were selected and finally analyzed for the positivity to the anti- Brettanomycesantibody. As evidenced, a subpopulation positive to the anti-Brettanomyces antibody was identified. The anti-Brettanomyces negative population represents the *S. cerevisiae* subset. It is interesting to note that the population identified with the anti-Brettanomyces antibody displayed FSC values lower than those observed for anti-Brettanomyces antibody negative yeasts, that, being *S. cerevisiae* cells, confirm the specificity of such a test (Figure 3b).

### 2.4. Flow Cytometry Identification and Count of B. bruxellensis in Wine Samples

**Figure 4:**
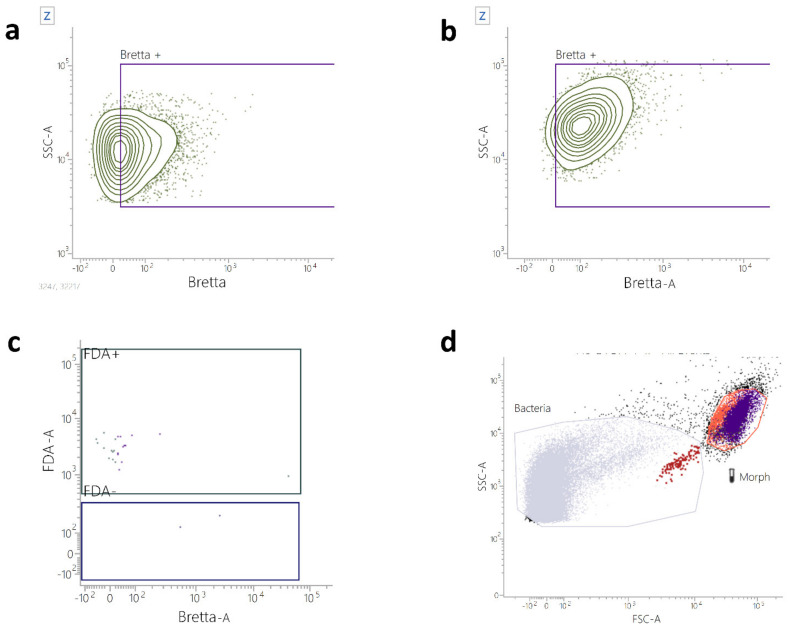
Flow Cytometry *B. bruxellensis* detection in wine samples. (**a**) The contour plot anti-Bretta/SSC-A represents a sample containing a mix of *B. bruxellensis* and *S. cerevisiae*. The plot shows the population of *B. bruxellensis* yeasts in the purple gate and the *S. cerevisiae* staining negative to the anti-Bretta antibody (at the left side of the purple gate). (**b**) The contour plot anti-Bretta/SSC-A represents a sample containing a pure population of *B. bruxellensis*. The plot shows the population of *B. bruxellensis* yeasts in the purple gate. (**c**) The dot-plot anti-Bretta/FDA shows the acquisition of a sterile wine sample. (**d**) The dot-plot SSC-A/FSC-A shows the scatter parameters of a sample containing a mix of *B. bruxellensis* and *O. oeni*. The grey gate contains the smaller detectable particles (*O. oeni*), while the orange gate contains the *B. bruxellensis* population of yeasts.

Once demonstrating the sensitivity and specificity of this method, we also analyzed three *B. bruxellensis* artificially contaminated and three naturally contaminated wine samples, using a sterile wine sample as a control. Figure 4b shows that, in a sample containing only *B. bruxellensis*, the whole yeast population stained positive to the anti-*B. bruxellensis* antibody (sample SW5). *B. bruxellensis* can be identified even in a mixed population of *B. bruxellensis* and *S. cerevisiae* (Figure 4a, sample S2), while when sterile wine is analyzed, no population of yeasts is stained by the kit (Figure 4c). Furthermore, by analyzing the scatter parameters, the yeast population can be distinguished from the bacteria (i.e., *Oenococcus oeni*, Figure 4d, sample SW6).

**Figure 5:**
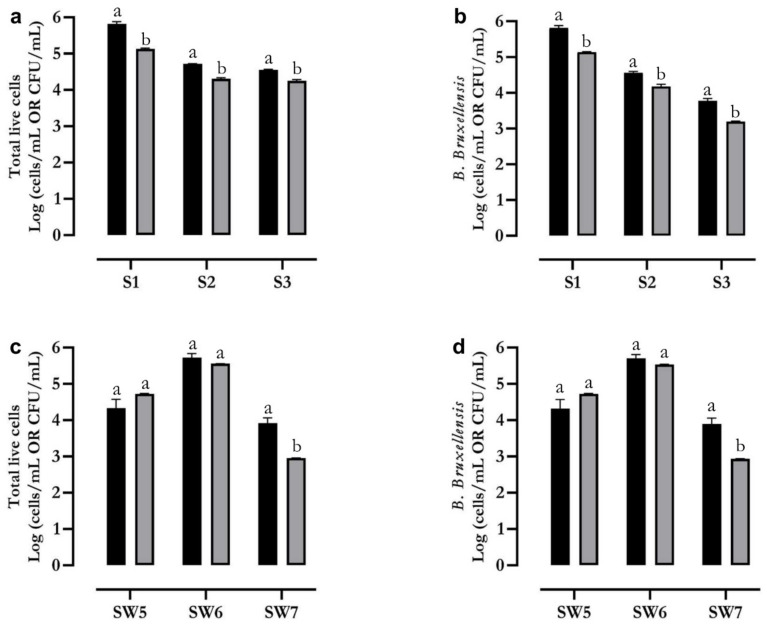
The concentrations of the total live cells (**a**) and *B. bruxellensis* (**b**) in artificially contaminated wine samples (samples 1–3) were carried out both by flow cytometry (black bars, FC, Log Cells/mL) and plate counts (grey bars, Log CFU/mL). The concentrations of total live cells (**c**) and *B. bruxellensis* (**d**) in naturally contaminated wine samples (samples 5–7) were carried out both by flow cytometry (black bars, FC, Log Cells/mL) and plate counts (grey bars, Log CFU/mL). Bars with different letters indicate significant differences (*t*-test, *p* < 0.05).

The concentrations of *B. bruxellensis* in wine samples was carried out by flow cytometry and paralleled to those obtained on the same samples (artificially or naturally contaminated) by plate counts (Figure 5a–d). The values obtained by plate counts were generally lower than those revealed by flow cytometry, confirming reported data showing that plate counts underestimate the concentrations of viable yeasts with respect to flow cytometry measurements, given that flow cytometry also allows the identification of viable, non-cultivable cells.

## 3. Discussion

*B. bruxellensis* contamination represents a problem for the wine industry [6,7,8]. For this reason, the rapid detection of *B. bruxellensis* contamination may be particularly useful for the adoption of appropriate strategies to avoid altered wine productions. The methods generally applied for *B. bruxellensis* detection are based on microbiological techniques. Unfortunately, those methods cannot identify viable but non-cultivable cells (VBNC) [1,16]. VBNC can instead be detected by molecular methods, based on the amplification of DNA and RNA fragments by the polymerase chain reaction (PCR). These methods are fast, specific, and sensitive [9]. The reverse transcriptase PCR (RT-PCR), employing an enzyme that synthesizes single-stranded DNA from RNA, is also able to discriminate between viable and non-viable cells. For this reason, it is largely employed. Nested-PCR is another molecular detection approach employed for *B. bruxellensis* detection. It uses two external and two internal primers, allowing the direct detection *B. bruxellensis* in wines without strain isolation [9]. More recently, a quantitative PCR by direct sampling (Cells-qPCR) has been used to detect and quantify yeasts in grape, must, and wine samples [24]. Cells-qPCR thus results a fast and sensitive technique. However, these molecular methods are more expensive than the plating techniques.

Recently, a flow cytometry kit (Bretta Test 80 tests, B80, Amarok Biotechnologies, Saint-Malo, France) for the identification of alive Brettanomyces yeasts in wines was optimized with the potential of overcoming the limitations linked to the abovementioned techniques. It must be underlined that in the past few years the use of polychromatic flow cytometry in the wine field greatly increased, given that it is a powerful technique allowing a rapid detection and enumeration of microbial populations in fermented food and during food production processes [25]. This technique, thanks to the use of probes (e.g., fluorescent molecules able to bind the DNA) and markers (e.g., fluorochrome-conjugated antibodies directed against specific microorganism markers), provides information about the presence of specific micro-organisms, about their physiological state and allows their enumeration in wine samples in a few hours [25]. Given that there are no publications evidencing the usefulness of this method, here we validated, for the first time, such a procedure. It is based on the identification of metabolically active *B. bruxellensis* cells, using the FDA and anti-Brettanomyces antibody. Our results demonstrated that the kit identifies homogeneous populations of metabolically active cells (FDA+), both in cell cultures and in wine samples (naturally and artificially contaminated). As already reported, with respect to the concentrations obtained by plate counts, flow cytometry also allows for identifying the population of viable, non-cultivable cells [22]. Therefore, microbiological techniques slightly underestimate yeast counts [26].

The specificity of the anti-Brettanomyces antibody provided by the kit was also demonstrated, given that we observed that different *B. bruxellensis* strains were successfully recognized by the antibody. We also observed that the kit stained the yeast cells in an efficient way even at low concentrations that produce natural contaminations (up to 10^5^ cells/mL). Of note, *B. bruxellensis* appears in the wine at concentrations of the order of 10^2^ cells/mL during the post-fermentation phase, while it can alter the wine characteristics when present at concentrations of the order of 10^3^ cells/mL [27]. It must also be underlined that, anyway, wines are characterized by the occurrence of a wide array of microbes [22]. The microbial dynamics depend on several factors, including the chemical characteristics of must/wine and its nutrients availability [28]. Moreover, *B. bruxellensis* is mainly present in red wines obtained from grape cultivars rich in ethyl-phenol precursors. In white wines, its occurrence is lower because pH is decreased with respect to that of red wines and therefore the SO_2_ is more effective in causing yeast death. Therefore, the possibility to rapidly monitor the concentrations of unwanted (i.e., *B. bruxellensis*) and wanted (i.e., *S. cerevisiae*) viable micro-organisms, colonizing the wines during the different fermentation and conservation stages is crucial to develop a more efficient production process [29]. For these reasons, here, we also demonstrated the ability of this flow cytometry method to identify and count *B. bruxellensis* in wine samples artificially and naturally contaminated by *B. bruxellensis* and *S. cerevisiae*. It must be underlined, anyway, that besides the fact that in wine samples the staining of *B. bruxellensis* is still effective, it resulted slightly weaker than in culture samples. This was possibly due to the ethanol presence, which is known to affect the antigen-antibody binding [30]. For these reasons, we strongly recommend involving a flow cytometry expert in the implementation of the test in a microbiological laboratory. Our results also are a proof of principle of the flow cytometry’s great potential in the enological field. The main advantage of the here presented method is that it rapidly detects yeast suspensions, and in particular Brettanomyces, it allows the obtainment of exact and accurate cell concentrations. Furthermore, this method allows identifying the exact number of live and dead cells, an evaluation that is not allowed by molecular methods. The possibility to develop other specific markers for the identification of different micro-organisms may completely change the paradigm of the enological productions.

## 4. Materials and Methods

### 4.1. B. bruxellensis Cell Cultures

Four strains of *B. bruxellensis* (Bb1, Bb2, Bb3 and Bb4), one *S. cerevisiae* and one *Pichia* spp. strain, belonging to the collection of Department of Agronomy, Food, Environmental and Forestry, University of Florence (Italy) were cultured separately at 30 °C for 48 h using 30 mL of liquid YPD medium (1% *w*/*v*) yeast extract, 2% (*w*/*v*) peptone, and 2% (*w*/*v*) glucose. Cell counts of viable cells were carried out using a Thoma chamber with methylene blue staining [31]. Phosphate Buffer Saline (PBS) solution was used to dilute the strain cultures in order to obtain samples at concentrations ranging from 10^2^ to 10^8^ cell/mL.

### 4.2. Wine Samples

Both wine (Sangiovese) samples artificially (Table 1) and naturally contaminated (Table 2) were analyzed. Wines were analyzed, in parallel, both by flow cytometry and plate counts, as summarized in Figure S1.

In particular, sterile wine samples, obtained by membrane filtration (0.45 µm porosity) were inoculated with the four *B. bruxellensis* strains mixed together: the above mentioned *S. cerevisiae* strain and *Pichia* spp., following the scheme reported in Table 1. Cell concentration of the single cultures was determined by Thoma chamber and used to achieve the final concentration.

Wine samples, naturally contaminated with different *B. bruxellensis* concentrations were also analyzed (Table 2). In some wine samples, *Oenococcus oeni* and *Pichia* spp. yeasts also occurred.

### 4.3. Flow Cytometry Analyses

Flow cytometry identification and *B. bruxellensis* counts were carried out at the Center for Advanced Studies and Technology (CAST, Chieti), by using the “Bretta test” kit (Kit Bretta Test 80 tests, B80, Amarok Biotechnologies, Saint-Malo, France) that contains an antibody recognizing Brettanomyces. Samples, diluted in PBS, were filtered with a 30 µm filter to desegregate cell clumps eventually present since cell clumps are not suitable for flow cytometry purposes. Samples were then centrifuged at 500× *g* at room temperature for 10 min. The supernatant was removed, and the pellet was stained as suggested by the manufacturer’s instructions. Briefly, samples were resuspended using Reagent 4 (200 µL) of the kit, stained with 10 µL of a rabbit polyclonal antibody directed against Brettanomyces antigens (anti-Brettanomyces antibody) and incubated for 20 min in the dark. Reagent 4 was used to wash the cells; samples were then centrifuged at 500× *g* for 10 min, resuspended in Reagent 4 (200 µL), and stained with 10 µL of fluorescein diacetate (FDA), when appropriate, and 10 µL of secondary anti-rabbit antibody PE-conjugated (Amarok Biotechnologies, Saint-Malo, France), or 1 µL of secondary antibody anti-rabbit Alexa-Fluor 633 conjugated (Thermo Fisher Scientific, Waltham, MA, USA). Samples were incubated for 15 min in the dark, washed, resuspended in Reagent 4, and acquired by Flow Cytometry (FACSVerse, BD Biosciences, San Jose, CA, USA, 3 lasers, 8 fluorescences). For each sample, at least 10,000 events/sample were recorded, and the staining was repeated and acquired three times. Cell concentrations were obtained using a volumetric count device (FACSVerse, BD Biosciences). Instrument performances, data reproducibility, and fluorescence calibrations were carried out by the Cytometer Setup & Tracking Module (BD Biosciences) [32,33]. The evaluation of non-specific fluorescence was obtained by acquiring fluorescence-minus-one controls, combined with the secondary antibody only [34,35]. Compensation was calculated using individually stained fluorescent samples. Data were analyzed using FACSuite v 1.0.6.5230 software (BD Biosciences). To verify the correct positioning of the gating on the dimensional dot-plots (SSC-FSC), MegaMixlus polystyrene beads of known size (Byocitex, Marseille, France) were used [36]. All parameters were analyzed using logarithmic or bi-exponential display modes. A pure culture of *S. cerevisiae* at a concentration of 10^5^ cells/mL was used as a negative control. Notably, given that the anti-Brettanomyces used by the kit is a rabbit polyclonal antibody, it is mandatory to optimize the staining (antibody titration, specificity verification, etc.) for each kit lot. FDA staining should also be optimized.

### 4.4. B. bruxellensis Counts

Plate count was carried out on different media and paralleled to flow cytometry counts. Different media were used: DBDM for B. bruxellensis [37], WL nutrient agar, Oxoid [38], with the addition of 2 g/L sodium propionate and 0.3 g/L streptomycin, for non-Brettanomyces yeasts, lactic bacteria MRS, ISO, agar, Oxoid [39], with the addition of 5 g/L fructose, 0.5 g/L cysteine, 2.5 g/L tomato juice broth, 6 g/L agar, and 0.05 g/L pimaricin, for lactic bacteria. Plates were incubated at 28 °C for 5–7 days until colonies developed and the results were expressed as colony forming units per milliliter (CFU/mL).

### 4.5. Statistics

Statistical analyses were performed using GraphPad Prism ver.8.0 (GraphPad Software Inc., La Jolla, CA, USA) and XLSTAT 2022 (Addinsoft, New York, NY, USA). Differences were tested using the Student’s *t*-test as appropriate. A *p*-value of <0.05 was considered statistically significant.

## 5. Conclusions

Altogether, our results provide evidence that polychromatic flow cytometry, together with the use of labeled antibodies, allowed us to rapidly detect *B. bruxellensis* yeasts. For these reasons, this technique has great potential for the detection of *B. bruxellensis* contaminates both in barrel-washing waters and in wines. The rapid execution of the test and the possibility to obtain cell counts of live and dead yeasts allows for effective interventions during fermentation, representing a major advantage with respect to plate count and molecular methods. Notably, flow cytometry costs are sustainable (slightly higher than plate count techniques) if the analyses are carried out by specialized laboratories. The need to send the samples to experts represents, anyway, a limitation for the wide application of flow cytometry in enology, even if the development of new strategies (such as “tele-flow cytometry”, which allows the telematic connection of the wine cellars with expert flow cytometry operators), would enable the application of these methods in enology and might open new perspectives for the improvement of wine production processes.

## Figures and Tables

**Table 1:** Wine samples are artificially inoculated with a pure culture of *B. bruxellensis* (1) and with mixed yeast cultures (2 and 3).

Wine Sample	Inoculated Cells	Concentration (Cells/mL)
S1	*B. bruxellensis*	8 × 10^5^
S2	*B. bruxellensis*	8 × 10^4^
	*S. cerevisiae*	8 × 10^3^
S3	*B. bruxellensis*	8 × 10^3^
	*S. cerevisiae*	8 × 10^3^
	*Pichia* spp.	8 × 10^3^

**Table 2:** Wine samples naturally contaminated by only *B. bruxellensis* or by *B. bruxellensis* and other yeast species or by *B. bruxellensis* and *O. oeni*.

Wine Sample	Inoculated Cells	Concentration (Cells/mL)
SW5	*B. bruxellensis*	(6.0 ± 0.3) × 10^3^
SW6	*B. bruxellensis*	(3.9 ± 0.1) × 10^4^
	*O. oeni*	(3.3 ± 0.3) × 10^3^
SW7	*B. bruxellensis*	(9.4 ± 0.5) × 10^2^
	*Pichia* spp.	(2.6 ± 0.7) × 10^2^

## Data Availability

Data will be available on reasonable request.

## Supplementary Materials

The following supporting information can be downloaded at: https://www.mdpi.com/article/10.3390/ijms232315091/s1.
