# Supplementary material for: Rapid Detection of Brettanomyces bruxellensis in Wine by Polychromatic Flow Cytometry
Source: Int J Mol Sci. 2022 Dec 1;23(23):15091. doi: 10.3390/ijms232315091 (PMC9740995; doi:10.3390/ijms232315091)
Supplement: Supplementary file 1 [file ijms-23-15091-s001.zip › ijms-1986458-supplementary.pdf]

International Journal of *Molecular Sciences*

MDPI

Supplementary

Rapid Detection of *Brettanomyces bruxellensis* in Wine by Polychromatic Flow Cytometry

Domenico De Bellis [1,2,3], Alessio Di Stefano [2], Pasquale Simeone [1,2,3,*], Giulia Catitti [1,2], Simone Vespa [1,2], Antonia Patruno [1], Marco Marchisio [1,2], Eleonora Mari [4], Lisa Granchi [4], Carlo Viti [4], Piero Chiacchiaretta [5,6], Angelo Cichelli [5], Rosanna Tofalo [7] and Paola Lanuti [1,2,3]

1 Department of Medicine and Aging Sciences, University "G. d'Annunzio" of Chieti-Pescara, 66100 Chieti, Italy

2 Center for Advanced Studies and Technology (CAST), University "G. d'Annunzio" of Chieti-Pescara, 66100 Chieti, Italy

3 FlowForLife Lab, spin-off, Center for Advanced Studies and Technology (CAST), University "G. d'Annunzio" of Chieti-Pescara, 66100 Chieti, Italy

4 Department of Agronomy, Food, Environmental and Forestry, University of Florence,

5 Department of Innovative Technologies in Medicine and Dentistry, "G. d'Annunzio" University of Chieti-Pescara, 66100 Chieti, Italy

6 Advanced Computing Core, Center for Advanced Studies and Technology—C.A.S.T, University "G. d'Annunzio" of Chieti–Pescara, Via Luigi Polacchi 11, 66100 Chieti, Italy

7 Department of Bioscience and Technology for Food, Agriculture and Environment, University of Teramo, Via R. Balzarini 1, 64100 Teramo, Italy

* Correspondence: simeone.pasquale@gmail.com

Citation: De Bellis, D.; Di Stefano, A.; Simeone, P.; Catitti, G.; Vespa, S.; Patruno, A.; Marchisio, M.; Mari, E.; Granchi, L.; Viti, C.; et al. Rapid Detection of *Brettanomyces bruxellensis* in Wine by Polychromatic Flow Cytometry. *Int. J. Mol. Sci.* **2022**, *23*, x. https://doi.org/10.3390/xxxxx

Academic Editor(s): Cristina Martínez-Villaluenga

Received: 7 July 2022
Accepted: 24 November 2022
Published: date

Publisher's Note: MDPI stays neutral with regard to jurisdictional claims in published maps and institutional affiliations.

Copyright: © 2022 by the authors. Submitted for possible open access publication under the terms and conditions of the Creative Commons Attribution (CC BY) license (https://creativecommons.org/licenses/by/4.0/).

International Journal of
Molecular Sciences

MDPI

Syntetic wines	Yeast inoculated	T0 Concentration (cells/mL)
Sample 1	B. *bruxellensis*	8 x 10^5
Sample 2	B. *bruxellensis*	8 x 10^4
	S. *cerevisiae*	8 x 10^3
Sample 3	B. *bruxellensis*	8 x 10^3
	S. *cerevisiae*	8 x 10^3
	Pichia spp.	8 x 10^3
**Naturally contaminated wines**	**Yeast inoculated**	**T0 Concentration (cells/mL)**
Wine Sample 5	B. *bruxellensis*	(6,0 ± 0.3) x 10^3
Wine Sample 6	B. *bruxellensis*	(3,9 ± 0.1) x 10^4
	O. *oeni*	(3,3 ± 0.3) x 10^3
Wine Sample 7	B. *bruxellensis*	(9,4 ± 0.5) x 10^2
	Pichia spp.	(2,6 ± 0.7) x 10^2

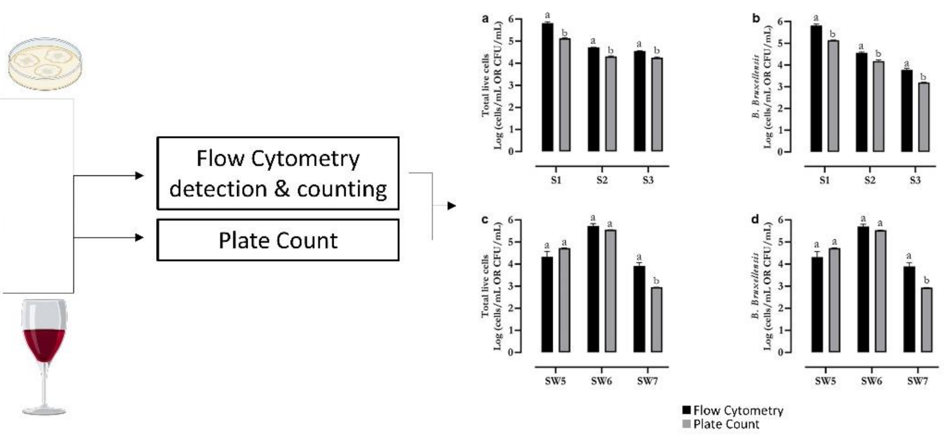

Figure S1. Wine samples artificially inoculated and naturally contaminated with B. bruxellensis, alone or in combination with others yeast species and bacteria, were analyzed in parallel by two different counting methods (polychromatic flow cytometry and plate count). Results, expressed as number of cells/mL were compared to validate the flow cytometry counting method.
